# Long-term effects and psychological adjustment: study protocol of a large register-based study on quality of life among survivors of hematological malignancies

**DOI:** 10.1186/s12885-017-3454-7

**Published:** 2017-07-12

**Authors:** Peter Esser, Katharina Kuba, Heide Götze, Anja Mehnert

**Affiliations:** Department of Medical Psychology and Medical Sociology, University Medical Center Leipzig, Philipp-Rosenthal-Str. 55, 04103 Leipzig, Germany

**Keywords:** Hematological malignancies, Long-term effects, Late effects, Survivorship, Health and wellbeing, Psychological adjustment

## Abstract

**Background:**

Both incidence and survival rates of hematological cancers are increasing, leading to a growing number of survivors with specific late and long-term effects. However, relevant research in physical, psychological and social aspects of quality of life is scarce. Existing literature shows that a considerable number of cancer survivors report a relatively high quality of life despite a variety of adverse and persistent symptoms. To date, the reasons for this phenomenon as well as moderating and mediating factors are widely unknown. Given these research gaps, we aim to investigate the different domains of quality of life among long-term survivors of hematological cancers and to identify factors predicting high quality of life.

**Methods/Design:**

This is a large cross-sectional study among hematological cancer survivors at a minimum of 3 years after diagnosis. We will collect 1000 survivors completing a set of self-report-questionnaires encompassing physical, psychological and social domains of quality of life. Participants are clustered in groups according to time since diagnosis and compared with each other. Furthermore, survivors will be compared with the general population. Factors predicting high quality of life will be identified via multiple regression analyses and structure equation modeling.

**Discussion:**

Our study will help to inform health care providers about the specific long-term burden among survivors with hematological malignancies. Identification of factors predicting high quality of life will help to develop adequate intervention strategies to enhance well-being in hematological cancer survivors. Our methodological advantages including the large sample as well as the assessment of different domains of quality of life will ensure novel and robust results. A limitation of the study is the cross-sectional design.

## Background

Fortunately, survival rates among hematological cancer patients are considerably improving [1, 2]. In a Europe-wide study among 6.7 million cancer patients, Hodgkin’s and non-Hodgkin-Lymphomas were among those types with the highest improvement in survival rates [3]. Given the rising incidence of hematological cancers in the industrialized countries [4], the health care systems are confronted with a growing population strained by specific adverse late or long-term effects.

The first step in developing adequate intervention strategies and survivorship care plans is to assess the specific medical and psychosocial needs of cancer survivors [5]. However, compared to other cancer types, only few studies exist assessing quality of life (QoL) in hematological cancer patients [6–8]. This is problematic, as hematological cancer types differ from other cancer sites in many aspects. Some types are classified to be chronic [9, 10], while others have a high risk for relapse, with rates up to 92% [11]. Moreover, the risk for developing a second malignancy is elevated up to 20 years after treatment [12, 13]. As many hematological cancer types are systemic, therapy is often more toxic and invasive compared to other malignancies. A treatment primarily used among this group is hematopoietic stem cell transplantation (HSCT) [14], which negatively affects a variety symptoms up to 10 years after HSCT [14–20]. All these features negatively impact the patients’ lives in many aspects. For example, hematological cancer patients have a three-fold increased risk for quitting work due to cancer when compared to colorectal cancer survivors [21].

Regarding change in QoL over time, some studies found time since diagnosis to have an effect on QoL [22, 23], while others did not [8, 24, 25]. Paradox seems the result by Miltény et al. [26] showing that survivors of Hodgkin’s Lymphoma with more than 20 years after treatment had a significant higher level in fatigue than patients under current treatment. Another finding is that different QoL domains seem to resolve or occur in specific time frames. For example, Syrjala et al. [19] showed that physical impairment among leukemia and lymphoma patients (baseline *n* = 319) treated with HSCT improves more rapidly than impairments in the psychological or psycho-social domains.

When comparing long-term survivors with non-cancer control groups, Wettergren et al. [27] revealed that QoL among survivors of Hodgkin’s Lymphoma (*N* = 121) at a mean of 14 years after diagnosis did not significantly differ from a control group. In another study among hematological cancer patients 10 years after HSCT, survivors (*N* = 137) reported more medical problems than controls, but did not differ in psychological health [18]. Two studies showed better physical functioning [22] and lower bodily pain [23] in Hodgkin’s Lymphoma survivors 10–15 years after transplantation than a comparison group from the general population. This surprising fact is supported by qualitative data based on cancer survivors between 6 and 18 years after HSCT, indicating that despite several impairments, the majority see themselves as relatively well [28]. Taken together, previous research is inconclusive and mostly based on HSCT patients, which reduces the generalizability of the findings.

Furthermore, the aforementioned results pose not only the question of whether, to what extent and when hematological cancer survivors experience late and long-term effects, but also *how* they adapt to them. In this context, Zebrack et al. [29] suggested that QoL might be partially explained by the cognitive frame or the meaning they attribute to the cancer experience. Another study by Lim et al. [30] among long-term survivors of leukemia and lymphoma (*N* = 53; ≥ 10 years after diagnosis) identified non-medical predictors of QoL such as satisfaction with social support or use of supportive care services. Other studies among cancer patients discussed the meaning of coping styles, self-efficacy or global appraisal of stress in predicting better adaptation and QoL [31–33]. Concepts linking clinical variables with QoL mediated by personality characteristics are also discussed outside the oncology setting [34]. More research is needed to identify factors influencing adjustment of long-term cancer survivors [29], which in turn could help to enhance QoL in cancer patients with adequate interventions strategies.

## Objectives

Taking into account research gaps, inconclusive results and highly selected samples in previous studies, our primary aim is to investigate long-term effects of physical, psychological and social domains of QoL among a large sample of hematological cancer survivors, starting from 3 years after diagnosis. This approach will allow us to present various dimensions of QoL at different phases following a hematological cancer diagnosis. Large comparison data from the general population will help to better estimate the burden of those patients. Beyond this descriptive scope, our second aim is to look for non-medical and non-physical factors moderating or mediating the relationship between medical/physical burden and subjective well-being. This will help to identify and to therapeutically address features that are predictive for high QoL despite adverse and long-standing consequences.

## Methods/Design

### Study design

This is a large cross-sectional study among hematological cancer survivors. Owing to chronicity as well as long treatment and rehabilitation periods among respective patients, we chose a minimum period of 3 years after primary diagnosis. All participants will fulfill a set of self-report-questionnaires, either paper pencil or online.

### Study participants

We will collect data of at least 1000 patients with malignant neoplasms of lymphoid, hematopoietic and related tissue (ICD-10: C81-C96). Further inclusion criteria are (i) minimum period of 3 years after diagnosis, (ii) minimum age of 18 years at time of diagnosis, (iii) maximum age of 85 at time of assessment, (iv) sufficient knowledge of the German language, (v) physical, psychological and cognitive ability for study participation and (vi) written informed consent. We seek to end with five relatively equal groups (*n* = 200 each) clustered in 3–5 years, 6–8 years, 9–11 years and 12–14 years and ≥15 years after first diagnosis.

### Recruitment

Collaborations with the Clinical Cancer Registry at the Cancer Center Leipzig and the Epidemiologic Cancer Registry of Schleswig-Holstein ensure access to contact information for eligible patients in two cancer registries. Trained personnel in the two institutions (the city of Leipzig and the federal state of Schleswig-Holstein) extract patients who both gave general permission to be contacted for research projects and fulfill our inclusion criteria. Eligible patients are then contacted by sending them a package containing (i) a letter in which they are asked to participate in the study (ii) a flyer with important study information, (iii) the questionnaire, (iv) a declaration of consent and (v) a stamped envelope. Participants fill in the questionnaire and the declaration of consent and send these documents back to the coordinating study center. Alternatively, patients can participate online by using the software LimeSurvey [35]. Patients who do not respond within the next weeks are reminded. In case they do not wish to participate, they are asked to report their reason for non-participation on a form enclosed in the reminding package.

**Fig. 1:**
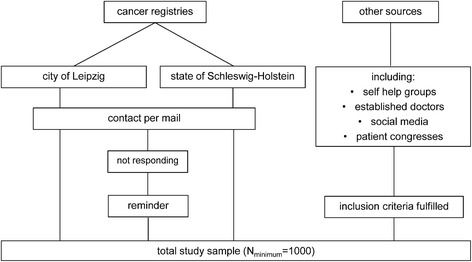
Recruitment procedure and sample composition

According to the tumor center of Leipzig, around 60% of all extracted (i.e. eligible) survivors will be deceased or cannot reached due to organizational reasons (e.g. change of name by marriage, move). To ensure our target sample size, we also collect participants from other sources, including social media, patient congresses, established doctors and self-help groups. An overview of our recruitment procedure and sample composition is given in Fig. 1.

### Bias control

Responders and non-responders will be analyzed in terms of important sociodemographic (such as age and gender) and medical characteristics (such as type of diagnosis and time since diagnosis). Significant group differences will be taken into account in both statistical models and interpretation of the findings. Furthermore, reported reasons for non-participation, e.g. organizational reasons or physical and psychological burden, will be evaluated to estimate other possible sample biases.

Finally, participants are assigned to two major groups, i.e. ‘cancer registry’ vs. ‘other sources’. Respective bias control will be ensured by either separate analyses or by including a group variable in multivariate analyses.

### Minimum sample size

The target sample size is based on the minimum number of patients in each of the five subsamples (3–5 years, 6–8 years, 9–11 years, 12–14 years and ≥15 years) that is necessary to identify predictors for high QoL.

As a first step, we estimated the expected amount of patients with relatively high QoL. For this purpose, we used a previous study among hematological cancer patients at a mean of 7 years after diagnosis (for further information see T3-sample in Esser et al. [36]). In detail, we calculated the percentage of patients with a QoL-score of the EORTC-QLQ-C30 [37] not less than one standard deviation below the German norm values [38]. This applied to around 60% of the patients. As a second step, we calculated the required sample size which is necessary to apply appropriate multiple regression analyses. For this a priori-computation, we applied G*Power 3.1 [39], using ‘Fixed Model, R^2^ increase’ [40]: Given a test power of 80% and an alpha-level of .05, a sample size of 114 is needed to determine an effect of f^2^ = 0.10 in multiple regression analyses that allow to test for three potential predictors when taking into account the three most important control variables (age, gender and diagnosis). Given that around 60% of the patients show high QoL and therefore can be used in such analyses, we need subsamples of *n* = 200 to ensure a minimum sample of *n* = 114 to apply appropriate multiple regression analyses. Consequently, the minimum total sample size was set at *N* = 5*200 = 1000.

### Comparison groups

To estimate the burden among the cancer survivors, we compare their results with norm values. Thanks to representative surveys organized by our institution with the assistance of a demographic consulting company (USUMA, Berlin, Germany), we have access to data sets containing large, nationwide and randomly selected samples among the general population. In detail, data is available for the EORTC-QLQ-C30 (*N* = 2448) [38], FLZ^M^ (*N* = 5036) [41], PHQ-9 (*N* = 5018) [42], GAD-7 (*N* = 5030) [43], NCCN Distress Thermometer (*N* = 2437; previously unpublished), F-SozU (*N* = 2507) [44] and PFB-K (*N* = 1390) [45].

### Measurements

The questionnaire was developed after extensive literature research and interviews with patients and experts (hematologists/oncologists). Instruments can be loosely divided in (i) sociodemographic and medical information, (ii) QoL in its different domains and (iii) premorbid/personality traits. In Table 1, every instrument is assigned to its respective domain. Below, detailed description can be found in order of assessment.

**Table 1:** Overview and categorization of instruments applied in the questionnaire

Domain	Issue	Measures
Patient characteristics	Sociodemographic	Internally developed
	Medical	Internally developed
Quality of life		
Physical	Symptoms; physical and cognitive functioning	EORTC-QLQ-C30 [37]
	Cancer-related fatigue	BFI [55]
	Cognitive functioning	AFI [68]
	Comorbidity	Comorbidity Index [76]
	Care level and disability	internally developed
	Late/long-term effects	internally developed
Psychological	Emotional functioning	EORTC-QLQ-C30 [37]
	General satisfaction	FLZ^M^ [47]
	Depressive symptoms	PHQ-9 [50]
	Anxiety symptoms	GAD-7 [50]
	General distress	NCCN Distress [52] Thermometer [52]
	Fear of progression	FoP-Q [54]
	Late/long-term effects	internally developed
Social	Role and social functioning; financial difficulties	EORTC-QLQ-C30 [37]
	Social and medical care need	SCNS-SF34 [70]
	Social support	F-SozU [44]
	Use of/satisfaction with social care	internally developed
	Use of/satisfaction with medical care	internally developed
	Employment and work ability/conditions	WAI [72]; internally developed
	Partnership, sexuality and fertility	PFB-K [45]; Geue et al. [75]; internally developed
	Late/long-term effects	internally developed
Premorbid/Personality Traits	Affectivity	PANAS [48]
	Coping styles	UCL-SF [57]
	Psychological flexibility	AAQ-II [60]
	Illness centrality	Wiebe et al. [63]; Helgeson et al. [63, 64]
	Changes in Self Concept	internally developed
	Health locus of control	MHLC [65]
	Health behavior	FEG [66]; report of Health Monitoring [67]; internally developed

*Note:* EORTC-QLQ-C30 and questions on late/long-term effects are assigned to all QoL domains due to multidimensionality/open response format

#### Sociodemographic and medical information

Relevant patient characteristics including gender, age, diagnosis according to ICD-10, date of diagnosis, types of therapy and previous cancers are obtained from the cancer registries, but also assessed in the questionnaire. Furthermore, patients are asked to report on disease status and complications in the course of the disease, partnership, children, living conditions, socioeconomic and job status as well as religiosity.

#### Quality of life (EORTC-QLQ-C30)

The European Organization for Research and Treatment of Cancer Quality of Life Questionnaire (EORTC-QLQ-C30) [37] is well-established across cancer sites and has been validated in German [46]. It contains 30 items, of which 28 are rated on a four-point Likert scale ranging from ‘not at all’ to ‘very much’ and can be clustered/assigned to five functioning scales (physical, role, cognitive, emotional and social), three symptom scales (fatigue, nausea, pain) and six one-item scales (dyspnea, sleeping problems, loos of appetite, constipation, diarrhea, financial problems). Additionally, general health and global QoL are rated on a seven-point Likert scale ranging from ‘very poor’ to ‘excellent’.

#### Satisfaction with life (FLZ^M^ – General Life Satisfaction)

The Questionnaire on Life Satisfaction (FLZ^M^) is developed and validated in German [47] and consists of two modules assessing general life satisfaction and satisfaction with health. For our study, we use the first module assessing general satisfaction with life, including health, income/financial security, occupation/work, housing/living conditions, family life/children, partner relationship/sexuality, friends/acquaintances and leisure time/hobbies. Each participant rates the subjective importance of each of these areas and, subsequently, the satisfaction with the respective domains. All items are rated on a five-point Likert scale ranging from ‘not important/unsatisfied’ to ‘extremely important/very satisfied’.

#### Affectivity (PANAS)

The Positive and Negative Affect Schedule (PANAS) [48] is validated in German [49] and contains 20 items assessing rather positive (active, interested, excited, strong, inspired, enthusiastic, proud, alert, determined, attentive) and rather negative emotions (distressed, upset, guilty, scared, hostile, irritable, ashamed, nervous, jittery, afraid). Participants are asked to rate how they feel in general, using a five-point Likert scale ranging from ‘not at all/very slightly’ to ‘extremely’.

#### Depressive (PHQ-9) and general anxiety disorder (GAD-7) symptomatology

The Patient Health Questionnaire (PHQ) [50] is validated in German [51] and assesses psychiatric disorders according to the DSM-IV criteria. For our study, we use the modules for depressive (PHQ-9; e.g. ‘little interest or pleasure in doing things’) and general anxiety disorder (GAD-7; e.g. ‘trouble relaxing’) symptomatology. The frequency of respective symptoms within the last 2 weeks is rated on a four-point Likert scale ranging from ‘not at all’ to ‘almost every day’. At the end of each module, we placed the overall item of the PHQ assessing difficulties in work, at home or in social context which can be attributed to one or more problems checked in the list. These two items are rated on a four-point scale ranging from ‘not difficult at all’ to ‘extremely difficult’.

#### General distress (NCCN distress thermometer)

The distress thermometer is a screener for general distress in cancer patients [52] and is validated in German [53]. The instrument consists of a single-item visual analogue scale ranging from 0 (no distress) to 10 (extremely distressed). A score of 5 or higher is interpreted as clinically significant.

#### Fear of progression (FoP-Q)

The Fear of Progression Questionnaire (FoP-Q) is developed and validated in German [54] and applicable for chronically ill patients. It encompasses affective reactions, partnership/family, work, loss of autonomy and coping. Since the four latter dimensions are assessed in other parts of our study, we restrict to affective reactions (e.g. ‘all types of little aches and pains make me anxious’). We further added two items, assessing the fear of cancer recurrence and late/long-term effects. All items are rated on a five-point Likert scale ranging from ‘never’ to ‘very often’.

#### Fatigue (BFI)

The Brief Fatigue Inventory (BFI) [55] is validated in German [56] and assesses fatigue in clinical populations. It first asks if the patient has felt unusually tired or fatigued in the last week. Patients then rate both the intensity of fatigue (at the moment, on average, strongest in the last 24 h) and its impact on general activity, mood, walking ability, normal work, relations with others and enjoyment of life. Items can be rated on a ten-point scale ranging from 0 (no fatigue/does not interfere) to 10 (as bad as you can imagine/completely interferes).

#### Coping styles (UCL-SF)

The Utrecht Coping List [57] measures how patients deal with stressful issues. We use a validated German abbreviation of this instrument [58], which has been applied in various populations [58, 59]. Its 23 items assess six dimensions, i.e. active problem solving, palliative behavior, avoidance behavior, search for social support, depressive reactions and comforting cognitions. Items can be rated on a four-point Likert scale ranging from ‘never or seldom’ to ‘very often’.

#### Psychological flexibility (AAQ-II)

Psychological flexibility was measured with the Acceptance and Action Questionnaire (AAQ-II) [60], which is translated and validated in German [61] and contains 7 items which can be rated on a seven-point Likert scale ranging from ‘never true’ to ‘always true’.

#### Illness centrality

Illness centrality measures the extent to which the cancer is central to a person’s identity. The construct is assessed based on questions originally developed by Wiebe et al. [62], which were further expanded and adapted for cancer patients [63, 64]. Patients are asked to answer 4 items (‘Being a cancer survivor is an important part of who I am’, ‘I think of being a cancer survivor when I think of who I am’, ‘Having had cancer is a small part of my life’, ‘I think a lot about having survived cancer’) on a six-point Likert scale ranging from ‘not at all true’ to ‘completely true’.

#### Changes in self-concept

To assess changes in concepts of the self after having had cancer, patients are asked to three times fill in blanks of the sentence ‘I am a _______ person because I had cancer.’ Answers will be analyzed qualitatively.

#### Multidimensional health locus of control (MHLC, Form A)

The validated Multidimensional Health Locus of Control (MHLC) [65] assesses the beliefs about one’s ability to control and to influence his or her own health. It is structured in three dimensions, i.e. internal, powerful others and chance. Out of two equal forms, we chose form A. It contains 18 items (e.g. ‘When I get sick, I am to blame’) that can be rated on a six-point Likert scale ranging from ‘strongly disagree’ to ‘strongly agree’. We translated this questionnaire into German.

#### Health behavior

Patients are asked about their drinking and smoking habits/history, physical exercise, relaxation techniques, regular intake of medicaments as well as weight and height (for calculating the BMI). The questions are loosely based on the German Questionnaire for the Assessment of Health Behavior (FEG) [66] and a report of the Federal Health Monitoring of Germany [67]. Most items can be rated on four-point Likert scales from ‘never’ to ‘daily’, others have to be affirmed and further specified (e.g. whether he/she smokes and if yes, how much).

#### Cognitive functioning (AFI)

The validated Attentional Function Index (AFI) [68] assesses cognitive functioning in common daily life activities focusing on attention and working memory. We translated this questionnaire into German. The first 9 items assess executive functioning (e.g. goal formulation, monitoring effective performance). The last 4 items measure behavioral and affective responses which go along with potential impairments in the executive functioning domains. All items can be rated on a visual analogue scale ranging from ‘not at all’ to ‘extremely well/a great deal’.

#### Social support (F-SozU)

The Social Support Questionnaire (F-SozU) [69] is developed in German and assesses perceived or anticipated social support. For our study, we use the validated short version (F-SozU K14) [44], containing 14 items. Items are rated on a five-point Likert scale ranging from ‘not true’ to ‘exactly true’.

#### Social and medical care need

The Supportive Care Needs Survey (SCNS-SF34) [70] assesses perceived needs in various domains and was validated in German [71]. To avoid redundancy, we extracted only those items which were not covered in other parts of the study, i.e. ‘Physical and daily living needs’ and ‘Psychological Needs’. We also added an item directly assessing the care need for physical problems. All items are rated on a four-point scale ranging from ‘no need’ to ‘high need’.

#### Use and satisfaction with social care

We use a module developed in our department, assessing whether the patient received support within the last month by either general practitioner/ward physician, nursing service, social workers, psychologist/psychotherapist, pastor/priest, self-help group, internet forum, relatives, friends or by any other source. In a second step, the patient rates the usefulness of the support on a five-point scale ranging from ‘not at all’ to ‘very helpful’.

#### Satisfaction with medical care

We ask the patients to evaluate the medical and psychosocial care received in the acute phase as well as in the after-care and whether they felt that physicians did take their concerns about side effects and late/long-term effects seriously. Questions can be rated on a seven-point Likert scale from ‘very dissatisfied’ to ‘very satisfied’.

#### Employment and work ability/conditions

We assess whether patients were employed at the time of diagnosis, whether they received rehabilitation or retraining and whether, how and to what extent they went back to work. Work ability/conditions are measured with adapted questions of the Work Ability Index [72], which was translated in German [73]. In detail, we assess work ability at the moment compared to the best work ability ever reached, sickness leave, the type of work (physical vs. mental) and the ability to work in each of the domains.

#### Partnership, sexuality and fertility

Satisfaction with partnership is assessed with item 10 of the short form of the German Questionnaire for diagnostic of partnership [74], called PFB-K [45]. Patients are further asked to estimate their satisfaction with their attractiveness and sexual life, perceived impairment in sexual joy by either physical or mental strain and what development they perceive compared to pre-diagnosis. The items are rated on five-point scales ranging from ‘extremely dissatisfied/never/much worse’ to ‘extremely satisfied/always/much better’. Items concerning fertility (completion of family planning at time of diagnosis, talks about fertility issues with the oncologist, impairment of fertility due to the cancer and its treatment) were taken from another cancer survivor study [75].

#### Comorbidity

We translated and adapted a validated comorbidity assessment instrument [76]. This questionnaire assesses if a patient has a certain condition and if yes, whether it interferes with his or her daily activities, ranging on a five-point scale from ‘not at all’ to ‘a lot’. To avoid redundancy, we summarized similar items (e.g. ‘coronary heart disease’ and ‘congestive heart failure’ was summarized to ‘heart diseases’). Furthermore, we added comorbidities which are typical for the hematological cancers and respective treatments, such as mucosal issues, liver disease, anemia and skin problems. We also assess occurrence of any psychiatric disorder at the moment and pre-diagnosis.

#### Care level and disability

We assess the occurrence of care need and disability and the official levels of disabilities.

#### Late and long-term effects

Finally, patients are directly asked to estimate whether and what conditions they attribute to the disease or treatment and how they are affected by those effects, ranging on five-point scale from ‘not straining at all’ to ‘very straining’.

### Statistical analyses

To investigated QoL over time, we will cluster the participants in groups with respect to the years since diagnosis and compare their means in relevant outcomes, e.g. via t-tests or chi-square tests. Those groups are then further compared with the general population. Effect sizes will be calculated to estimate the magnitude of significant effects. All analyses will be controlled for important variables such as gender, age and diagnosis.

For our aim to identify factors predicting patients to show relatively high QoL, we will apply multiple regression analyses. For more complex hypotheses, e.g. on moderating or mediating factors, we will apply structure equation modeling.

Additionally, our sample size and comprehensive assessment enables the application of confirmatory factor analyses and investigation of convergent/discriminant validity. Therefore, we will validate our German translations of the AFI [68] and the MHLC [65]. Furthermore, we will compare the patients from the cancer registry with the patients recruited from other sources. Such an investigation will be of great importance in interpreting results of the growing number of research based exclusively on patients recruited via social media.

## Discussion

To date, long-term data on QoL of hematological cancer patients is very scarce and mostly based on HSCT patients, thereby limiting generalizability of results. We conduct a relevant study featuring several methodological advantages ensuring novel results. First, we use a very large dataset, enabling us to stratify in well-defined groups with respect to time since diagnosis and thus to investigate QoL including physical, psychological and social aspects at different phases post-diagnosis. Second, access to large data sets of the general population in the most important outcomes will help to extract control groups perfectly matched by age and gender. Third, patient recruitment from two cancer registries in different parts of Germany improves generalizability. Fourth, the registries ensure correct information in the most important medical and sociodemographic variables and additionally allow for responder analyses. Finally, the sample size allows for applying methods to detect underlying processes that predict QoL. A limitation of the study is the cross-sectional design.

Taken together, our study will help to inform health care providers about the specific long-term burden among survivors with hematological malignancies and to develop adequate intervention strategies in order to heighten QoL in this specific patient group.
